# Hydrogen sulfide-sensitive Chitosan-SS-Levofloxacin micelles with a high drug content: Facile synthesis and targeted *Salmonella* infection therapy

**DOI:** 10.3389/fmicb.2022.1088153

**Published:** 2022-12-22

**Authors:** Chunbo Lu, Wenhui Lu, Xiantao Qin, Shuyi Liang, Congmin Niu, Jiayi Guo, Yujie Xu

**Affiliations:** ^1^Key Laboratory of Biology, School of Bioscience and Technology, Weifang Medical University, Weifang, Shandong, China; ^2^Shaanxi Key Laboratory of Natural Products and Chemical Biology, College of Chemistry and Pharmacy, Northwest A&F University, Yangling, Shaanxi, China

**Keywords:** antibiotic delivery systems, chitosan, hydrogen sulfide, micelles, targeted delivery

## Abstract

The delivery system of antibiotics plays an important role in increasing the drug efficacy and reducing the risks of off-target toxicities and antibiotic resistance. The pathophysiology of bacterial infections is similar to that of tumor tissues, but only a few delivery systems have been able to target and release antibiotics on demand. Herein, we designed and developed a robust Chitosan-SS-Levofloxacin (CS-SS-LF) micelles for targeted antibiotic delivery, in which disulfide bond can be reduced by hydrogen sulfide (H_2_S), a typical product of *Salmonella*, and subsequently released antibiotic to eradicate *Salmonella* infection. CS-SS-LF micelles showed uniform size and sharp response to H_2_S. Compared with levofloxacin alone, these micelles possessed a better capacity in disrupting *Salmonella* biofilms and reducing bacterial burden in organs. The H2S-sensitive CS-SS-LF micelles might enable a new way to address bacterial infections.

## 1. Introduction

Over the past few decades, the world has been plagued by microbial contamination. Humans are susceptible to many types of bacteria that can cause serious illness and even death (Francolini et al., 2004; El-Refaie et al., 2007). Among them, *Salmonella enterica* is one of the most prevalent bacterial pathogens. It is a Gram-negative facultative intracellular pathogen that crosses the intestinal barrier and is taken up by phagocytes, where it is able to reproduce and spread throughout the body (Prost and Riemann, 1967; Barton et al., 2011). *Salmonella* can cause serious infections, especially in children, and people with weakened immune systems (Christopher et al., 2001). Therefore, it is particularly important to further strengthen the surveillance and control of *Salmonella*.

As one of the most effective types of drugs for treating *Salmonella* infection, antibiotics have decreased mortality and morbidity rates and saved lives in countless cases (Nichterlein et al., 1998). In spite of this, antibiotics with low bioavailability lack specificity and because they are quickly metabolized and excreted by the circulatory system before reaching the infection site (Ghosh et al., 2016). As a result of overuse of antibiotics, antibiotic resistance emerges, reducing the effectiveness of clinical antibacterial agents. This poses a serious risk to public health (Chambers and Deleo, 2009; Stryjewski and Corey, 2014; Silva et al., 2016). New antibacterial solutions are urgently desired. The development of antibiotic delivery systems is a convenient way to reduce off-target toxicity and resistance to antibiotics while optimizing their efficacy and lifespan (Ghosh et al., 2016; Ning et al., 2018).

Drug release strategies utilizing individual microenvironments have gained widespread attention for treating a variety of diseases, including cancer (Ning et al., 2018), diabetes (Yannan et al., 2009), and bacterial infectious disease (Meng-Hua et al., 2012a). Recently, nanoparticle-based drug delivery showed its potential to solve bacterial infections (Alvarez-Lorenzo et al., 2016; Zaidi et al., 2017; Gao et al., 2018), which could enhance antibiotic targeting and eliminate premature drug release through controllable triggers that respond to the microenvironment, such as low pH (Chen et al., 2018), bacterial secretions (Dissaya et al., 2011), and enzyme overexpression (Meng-Hua et al., 2012b). Among these delivery systems, redox-responsive materials seem to be an attractive strategy, as some bacteria, such as *Salmonella*, are known to produce hydrogen sulfide (H_2_S) at infection sites (Lin et al., 2014).

A naturally occurring cationic polysaccharide, chitosan (CS) exhibits good biocompatibility, biodegradability, non-cytotoxicity, and low immunogenicity. As a kind of promising natural biomaterial, the research and application of CS in the field of biology and medicine arouse more and more emphasis (Hudson, 2003; Wang et al., 2019). Furthermore, as an antioxidant and antibacterial biopolymer, CS is effective against both Gram-negative and Gram-positive bacteria (Younes et al., 2014; Hajji et al., 2015).

**Figure 1:**
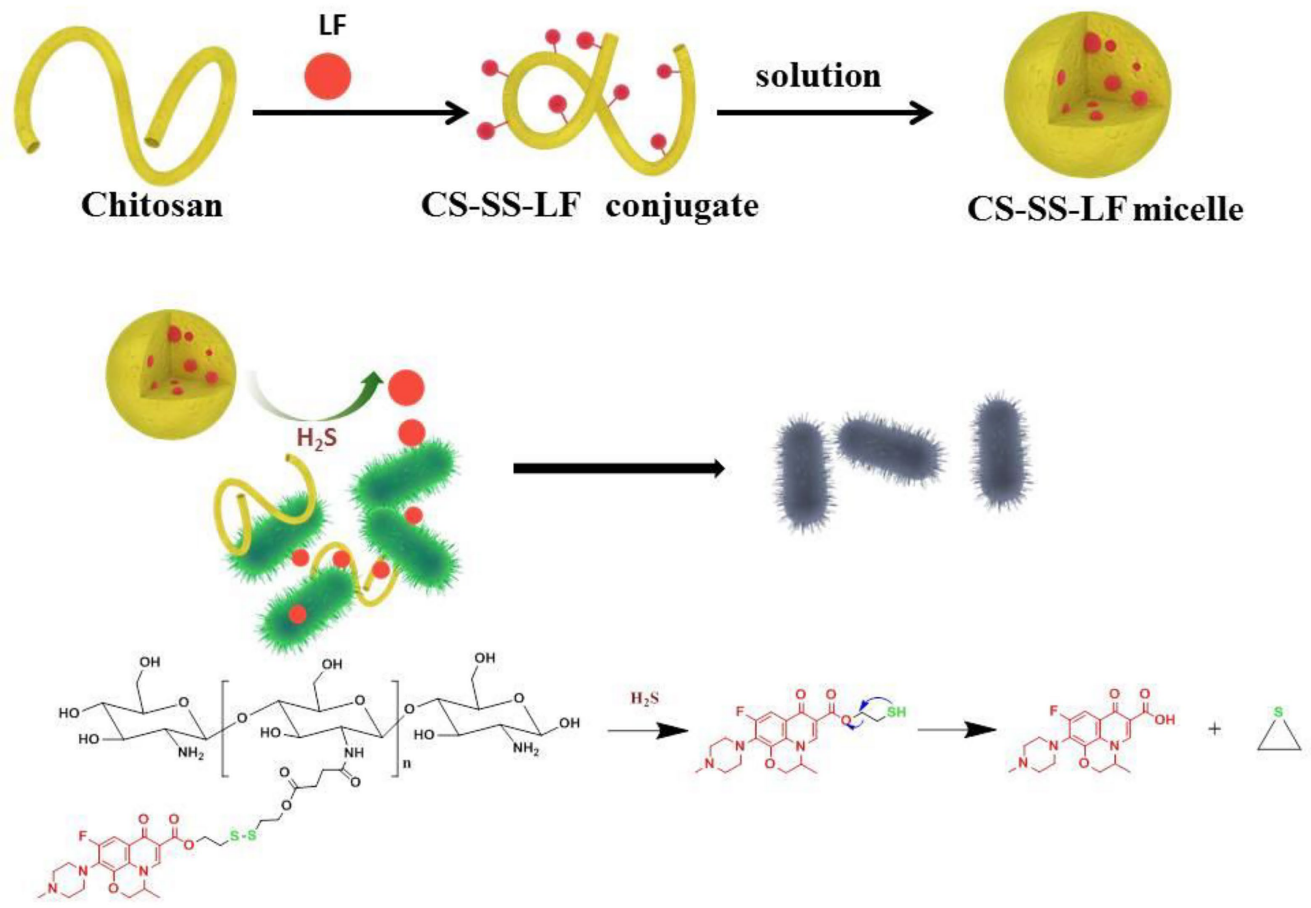
Diagram illustrating the antibacterial properties of CS-SS-LF micelles. Upon *Salmonella* infection, H_2_S actuates the cleavage of redox-sensitive triggers, resulting in the disintegration of micelle assemblies and release of intact antibacterial LF drug in active form.

Herein, we designed and developed a robust H_2_S-sensitive Chitosan-SS-Levofloxacin (CS-SS-LF) micelles for targeted and efficacious treatment of *Salmonella* infection (Figure 1). An antibiotic commonly used in the treatment of abdominal infections, levofloxacin (LF), was selected as the model antibiotic, as it is a broad-spectrum antibiotic that is active against both Gram-negative and Gram-positive bacteria. CS readily binds to negatively charged bacterial matrixes since it is a polycationic polysaccharide (Zhang et al., 2013). Taking clues from the above, we hypothesized taking advantage of the disulfide linkage, CS-SS-LF micelles can be triggered to disassemble by H_2_S produced by *Salmonella*, and simultaneously release antibiotic for antibacterial therapy. We demonstrated a proof-of-concept for designing CS-SS-LF micelles that would allow target therapy of specific bacteria, which may be a more effective way to utilize antibiotics for treating bacterial infections (Pal et al., 2016).

## 2. Results and discussion

### 2.1. Synthesis and characterization of Chitosan-SS-Levofloxacin

**Figure 2:**
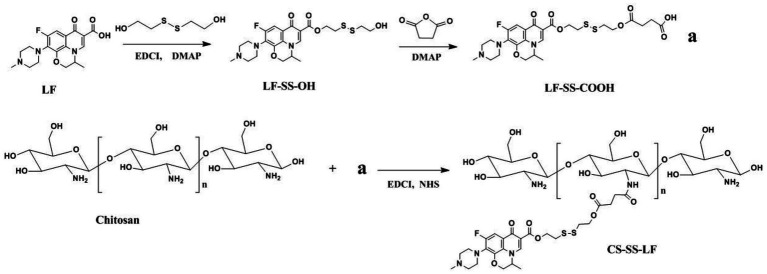
A synthetic route for CS-SS-LF conjugate.

**Figure 3:**
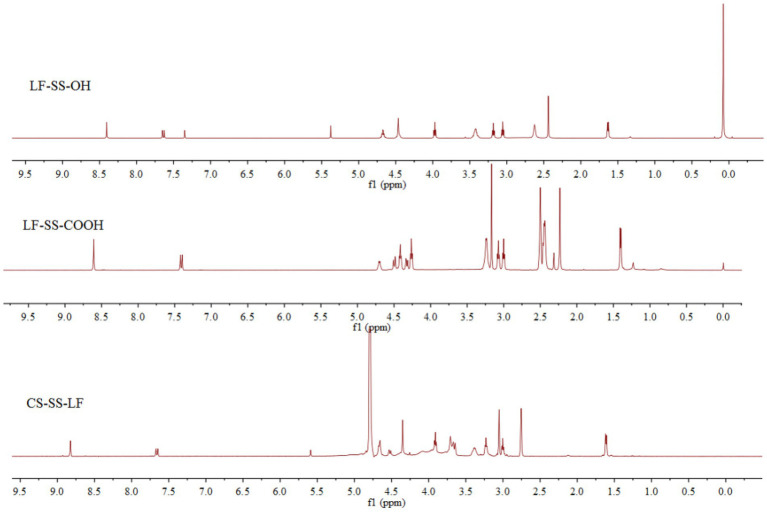
^1^H NMR spectra of LF-SS-OH, LF-SS-COOH and CS-SS-LF.

**Figure 4:**
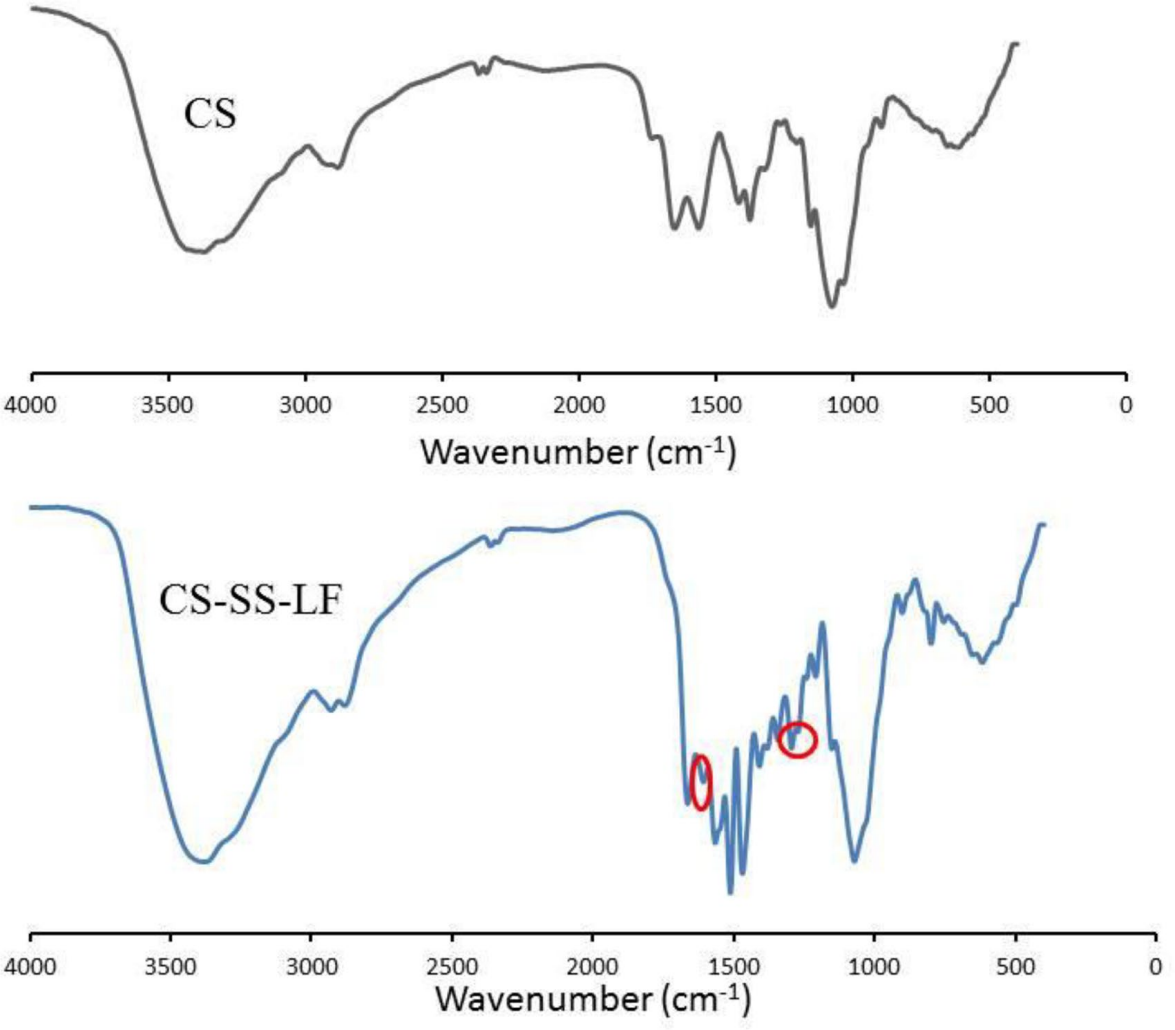
FTIR spectra of CS-SS-LF and CS.

Following the synthetic route shown in Figure 2, CS-SS-LF was synthesized. ^1^H NMR and FTIR confirmed the formation of the CS-SS-LF conjugate. As indicated in Figure 3, ^1^H NMR of the newly synthesized compound was in full agreement with the proposed structure. The signals of the phenyl hydrogen group and the vinyl hydrogen of LF moiety were observed at 8.82 ppm and 7.66 ppm, respectively. The typical signals of the methylene hydrogen were detected around 3.23–3.00 ppm, which indicated the formation of ester moiety. Moreover, the construction of the acylamide moiety was confirmed by the signal around 5.59 ppm, which corresponded to the hydrogen of the-CONH-group. The typical skeletal signals of CS were appeared around 4.12–3.64 ppm. The above appeared chemical shifts indicated the successful formation of the CS-SS-LF conjugate. The connection was also verified using FTIR analysis. As shown in Figure 4, as compared to those of CS, the new peaks of CS-SS-LF appeared at ~1294 cm^−1^ and 1620 cm^−1^, which assigned to stretching of amines and aromatic C-C (Jalvandi et al., 2017). As measured by UV–vis at 295 nm, the drug content of CS-SS-LF was 21.5 wt%, indicating synthesis success.

### 2.2. Characterization of Chitosan-SS-Levofloxacin micelles

**Figure 5:**
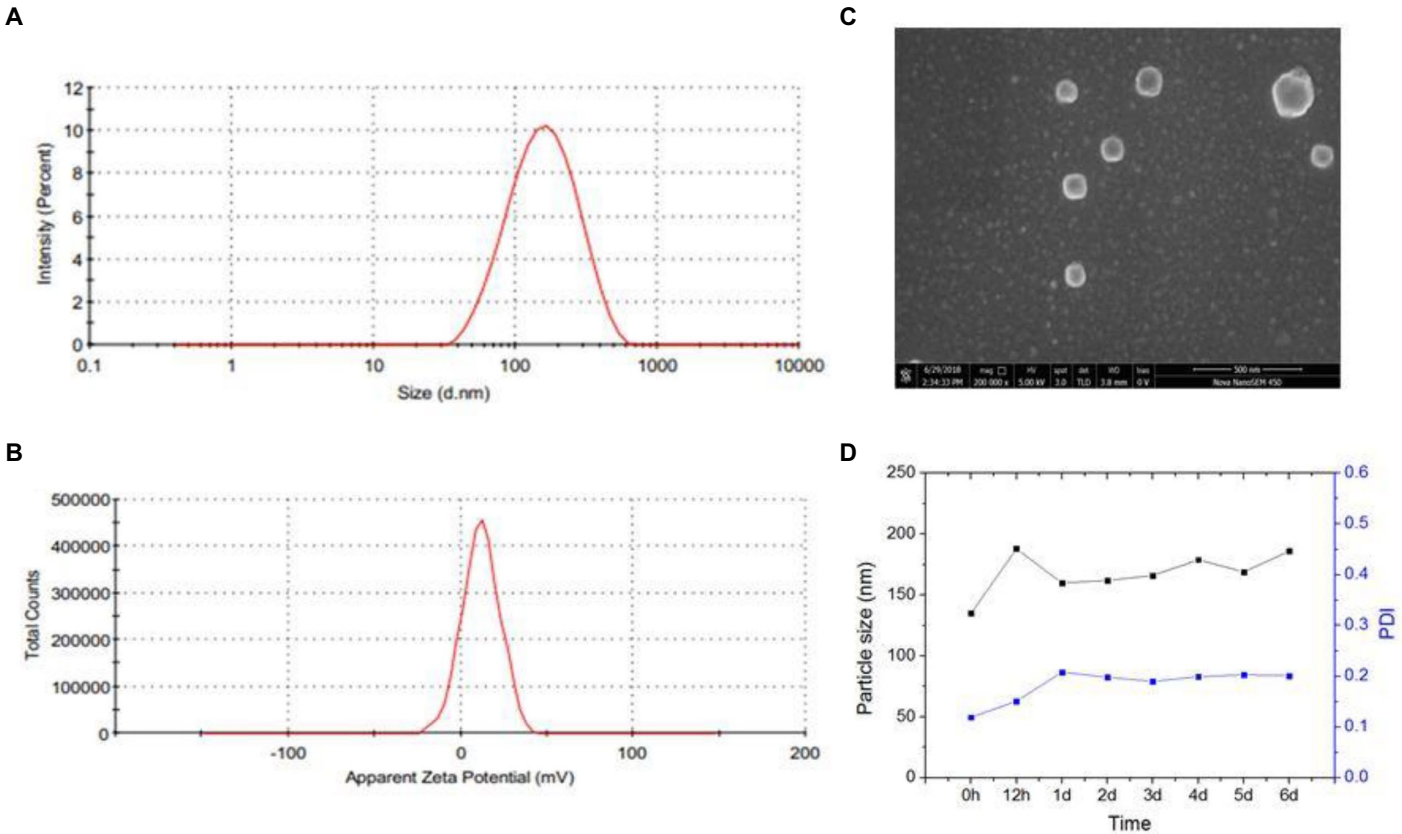
Characterization of CS-SS-LF micelles. **(A)** CS-SS-LF micelles were analyzed by DLS for size distributions. **(B)** CS-SS-LF micelles were analyzed by DLS for zeta potential. **(C)** SEM image of CS-SS-LF micelles. **(D)** Changes of diameter and PDI of CS-SS-LF micelles for 6 days at room temperature.

The amphiphilic CS-SS-LF conjugates readily formed micelles by self-assembling in aqueous medium. The morphology and size distribution of CS-SS-LF micelles were studied using scanning electron microscopy (SEM) and dynamic light scattering (DLS). Based on DLS measurements, CS-SS-LF micelles had a diameter of approximately 130 nm (Figure 5A) and a surface charge of 11.7 mv (Figure 5B), indicating that they contained cationic amine groups. As demonstrated by SEM in Figure 5C, the CS-SS-LF micelles displayed spherical morphology with approximately 120 nm in diameter. The stability of colloids is one of the most important aspects of nanoscale drug delivery systems. DLS measurements showed that CS-SS-LF micelles remained unchanged in diameter and polydispersity index for 6 days (Figure 5D). These results demonstrated that amphiphilic CS-SS-LF conjugates are excellent drug carriers since they can self-assemble into micelles.

### 2.3. *In vitro* drug release behavior

**Figure 6:**
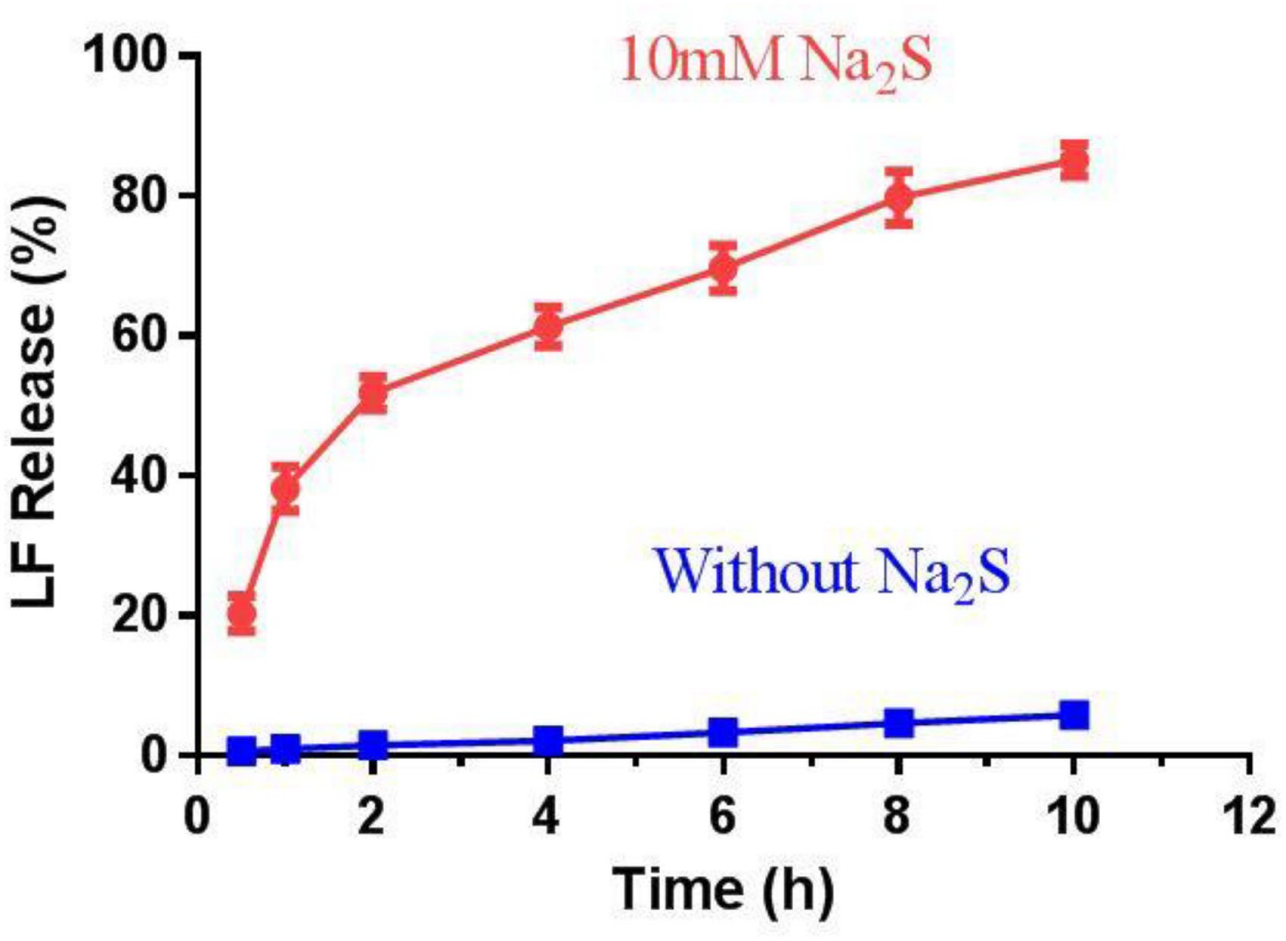
The release behavior of drugs from CS-SS-LF micelles *in vitro*. The release studies were conducted at pH 7.4 and 37°C with or without Na_2_S, respectively.

The trigger effect of H_2_S was further investigated by incubating CS-SS-LF micelles with or without 10 mM Na_2_S. As shown in Figure 6, about 55% drug was released in 2 h and reached 85%in 10 h with Na_2_S treatment. In contrast, less than 20% of the conjugated LF was released in PBS from CS-SS-LF micelles. As the disulfide bond being cleaved by H_2_S, rapid release occurs (Pal et al., 2016). The sustained retention is advantageous in micelles delivery because it prevents leakage of the drug prior to reaching the target site and ensures its delivery in larger quantities to be released at the infection site. According to these results, CS-SS-LF micelles exhibited high stableness under physiological conditions and can be used for targeted release.

### 2.4. Bactericidal effect of Chitosan-SS-Levofloxacin micelles on planktonic bacteria

To investigate the antimicrobial specificity of CS-SS-LF micelles, three strains (*Salmonella*, *P*. *aeruginosa*, and *S*. *aureus*) were selected as the model bacteria to qualitatively analyze the MIC of H_2_S-sensitive micelles, and the results were presented in Supplementary Table S1. It is apparent that the MIC of CS-SS-LF micelles against *P*. *aeruginosa* and *S*. *aureus* were significantly higher compared with that of free LF, marking that the nanodrug basically lost its antimicrobial activity against these two strains. However, the MIC of CS-SS-LF micelles against *Salmonella* was only slightly elevated (0.4 μg/mL vs. 0.25 μg/mL of net LF), revealing the high sensitivity and specificity of the modified micelles against *Salmonella*.

**Figure 7:**
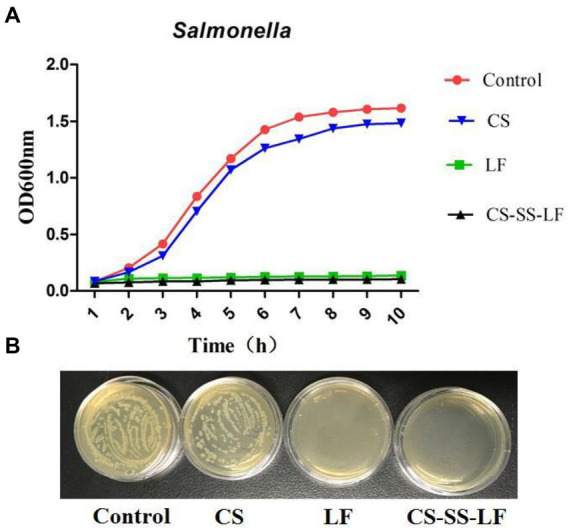
**(A)** The growth curves of *Salmonella* were determined by culturing bacteria in TSB containing CS-SS-LF micelles (80 μg/mL, [LF] = 17.2 μg/mL), CS (80 μg/mL) or LF (18 μg/mL). **(B)** CFU counts of *Salmonella* (10 h) after 10^6^-fold dilutions.

The bactericidal activity of CS-SS-LF micelles and LF were tested with *Salmonella* at different concentrations. As shown in Figure 7, cultured *Salmonella* bacteria in TSB containing CS-SS-LF micelles, equivalents of CS or LF, were analyzed for their growth curves. According to the results, CS-SS-LF micelles (80 μg/mL) inhibited the growth of *Salmonella* cells effectively compared to the blank control. By contrast, CS treatment had a less effective effect on inhibiting bacterial growth. Meanwhile, 10 μL samples (10^6^ dilution) were cultured on Petri dishes for 10 h and then the CFU were counted. Based on these results, CS-SS-LF micelles demonstrated ability in suppressing planktonic *Salmonella* growth.

### 2.5. Antibiofilm activities of Chitosan-SS-Levofloxacin micelles

**Figure 8:**
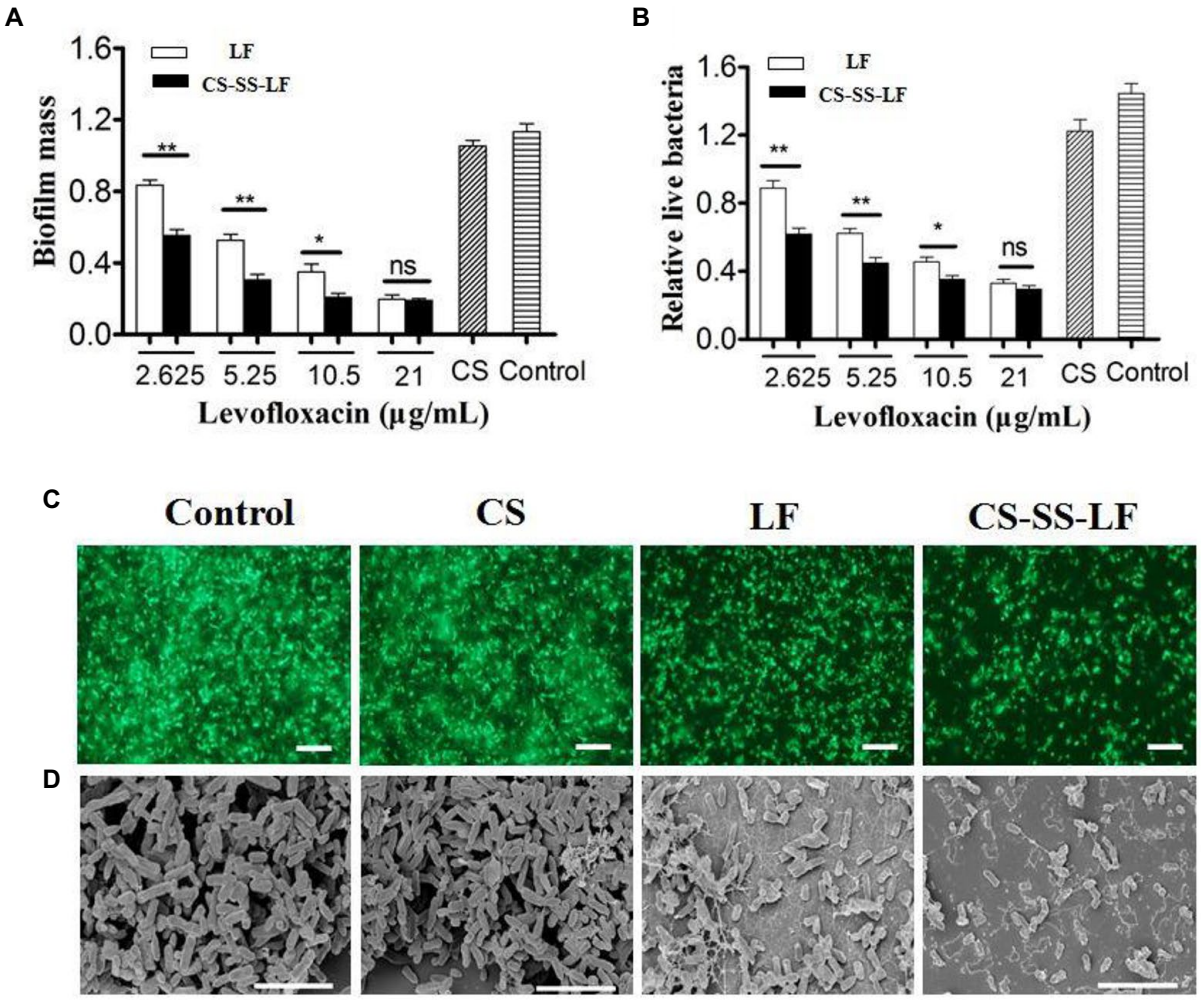
Antibiofilm activity of CS-SS-LF micelles. Crystal violet assay **(A)** and MTT assay **(B)** of *Salmonella* biofilm with CS, LF, and CS-SS-LF. Biofilms treated with CS-SS-LF are shown in the black bar, while those treated with LF are depicted in the white bar. **(C)** Fluorescence images of *Salmonella* biofilm, scale bar represents 10 μM. **(D)** SEM images of *Salmonella* biofilm, scale bar represents 5 μM. (*p < 0.05; **p < 0.01; ns: not significant).

As an opportunistic human pathogen with Gram-negative status, *Salmonella* is commonly employed as a biofilm model. *Salmonella* biofilms from established cultures were treated with different concentrations of CS-SS-LF micelles (12.21, 24.42, 48.84, 97.and 68 μg/mL) for 24 h to evaluate CS-SS-LF micelles^,^ disruption properties. It was determined that CS-SS-LF micelles were more effective at destroying live cells at all concentrations tested than LF alone (Figures 8A, B).

As shown in Figure 8C, *Salmonella* biofilms exposed to CS-SS-LF micelles for 24 h exhibited fewer scattered cell aggregates than individual reagents, as evidenced by visualization of biofilms by fluorescence microscopy.

*Salmonella* biofilms were treated with different samples and their apparent morphologies were assessed using SEM. As indicated in Figure 8D, biofilms that were treated with TSB have clearly intact bacteria cells with well-defined shapes and well-organized architectures. While some bacteria on the surface of the biofilms were destroyed by the CS treatment, intact cells and obvious aggregates were still visible. Surface roughness, cellular deformation, and cytoplasm leakage of bacteria were observed in LF and CS-SS-LF micelles groups. Noun broken bacterial cells were found in treated biofilms containing CS-SS-LF micelles.

Taken together, these results demonstrated that polycationic properties enabled CS-SS-LF micelles to be more effective in eliminating *Salmonella* biofilms than single drug.

### 2.6. *In vitro* cytotoxicity studies

**Figure 9:**
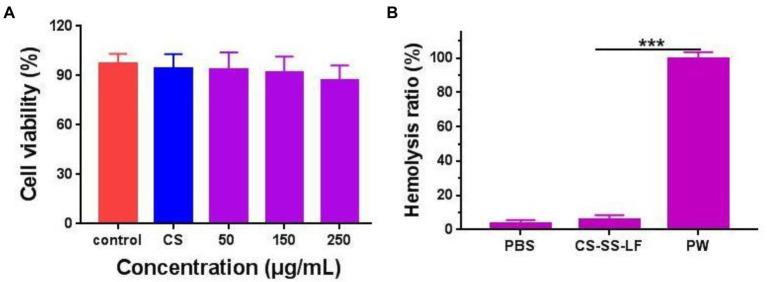
**(A)** Relative viabilities of RAW 264.7 cells after being exposed to CS-SS-LF micelles with different concentrations for 24 h. **(B)** The evaluation of the blood compatibility of CS-SS-LF micelles. (***p < 0.001).

For the MTT assay (Figure 9A; Supplementary Figure S1), cells were incubated with various concentrations of CS-SS-LF micelles for 24 h. We found that over 85% of the incubated cells remained viable after 24 h incubation even under the highest concentration of CS-SS-LF micelles at 250 μg/mL. In addition, the blood compatibility of CS-SS-LF micelles was estimated *via* a red blood cell hemolysis assay *in vitro*. As shown in Figure 9B, 250 μg/mL concentration in CS-SS-LF micelles group display no obvious in hemolysis of RBCs. The biocompatibility of these micelles further supported their potential as effective targeting vehicle for drug delivery.

### 2.7. Chitosan-SS-Levofloxacin micelles promoted bacteria clearance *in vivo*

**Figure 10:**
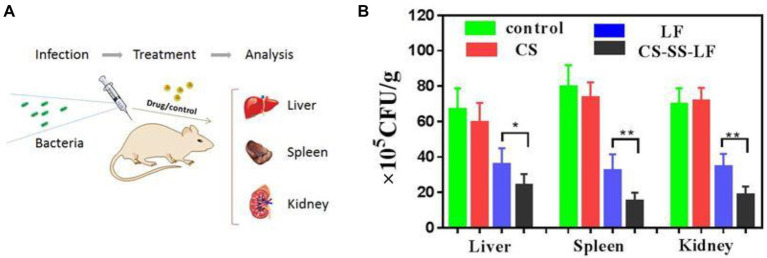
**(A)** An illustration of the *in vivo* experimental design based on a model of acute infection. **(B)** A statistical chart of the number of bacteria in the spleen, liver and kidney under CS-SS-LF treatment and control group treatment. (*p < 0.05; **p < 0.01; ***p < 0.001).

Test mice were infected with *Salmonella* (10^6^ CFU/mouse) and given therapy 24 h after infection. As indicated in Figure 10, liver, spleen, and kidney viable counts decreased when CS-SS-LF micelles (10 mg/Kg) and LF were treated by intraperitoneal injection. In addition, compared with LF, the CS-SS-LF micelles displayed better bacteria clearance in spleen and kidney. Study results showed that mice treated with CS-SS-LF micelles experienced decreased *Salmonella* infection in the intraperitoneal cavity. The strategy could serve as a useful tool in developing new therapies for *Salmonella*-associated infections.

## 3. Materials and methods

### 3.1. Materials

Chitosan (~10 kDa), MTT 3-(4, 5-dimethylthiazol-2-yl)-2, 5-diphenyl tetrazolium bromide, Succinic anhydride, 4-(dimethylamino) pyridine (DMAP), 1-(3-dimethylaminopropyl)-3-ethylcarbodiimide hydrochloride (EDC), Levofloxacin (LF), Dichloromethane (DCM) 2-Hydroxyethyl disulfide, N-hydroxysuccinimide (NHS) were obtained from Sigma Co. (St. Louis, MO, United States). Gibco Ltd. (Grand Island, NY, United States) provided the Roswell Park Memorial Institute (RPMI) 1,640 medium, while Hyclone (Logan, UT, United States) provided the fetal bovine serum (FBS).

Prof. Wang (College of Life Sciences, Northwest A&F University) generously donated *Salmonella typhimurium* (SL1344) and Macrophages (RAW264.7). We obtained Kunming mice from Pengyue Experimental Animal Breeding (Jinan, China).

### 3.2. Preparation of Chitosan-SS-Levofloxacin

#### 3.2.1. Synthesis of LF-SS-OH

LF-SS-OH was synthesized by esterification reaction in the presence of EDC and DMAP. Briefly, LF (723 mg, 2 mmol), EDC (422 mg, 2.2 mmol), DMAP (25 mg, 0.2 mmol) and 2-Hydroxyethyl disulfide (340 mg, 2.2 mmol) were taken in 40 mL anhydrous DCM in N2 atmosphere. At room temperature, the reaction mixture had been stirred for 48 h. After washing with sodium carbonate solution, the organic phase was dried with anhydrous sodium sulfate. A silica gel column chromatogram was conducted to obtain pure LF-SS-OH. LF-SS-OH was collected as a white powder with yield of 71.5%. ^1^H NMR (CDCl3-d1, 500 MHz): δ/ppm = 8.31 (s, 1H), 7.55 (d, J = 12.5 Hz, 1H), 5.29 (s, 1H), 4.62–4.56 (m, 2H), 4.44–4.33 (m, 3H), 3.88 (t, J = 6.0 Hz, 2H), 3.37–3.29 (m, 4H), 3.09 (t, J = 6.5 Hz, 2H), 2.97 (t, J = 6.0 Hz, 2H), 2.55–2.52 (m, 4H), 2.35 (s, 3H), 1.55 (d, J = 6.5 Hz, 3H).

#### 3.2.2. Synthesis of LF-SS-COOH

Succinic anhydride (150 mg, 1.5 mmol) and DMAP (15 mg, 0.12 mmol) were added to LF-SS-OH (598 mg, 1.2 mmol) solution in 30 mL anhydrous DCM. Stirring at room temperature under nitrogen for 12 h was performed on the mixture. Water was used to wash the reaction mixture, and the organic part was evaporated. A silica gel column chromatogram was conducted to obtain pure LF-SS-COOH. LF-SS-COOH was collected as a white power with yield of 81.9%. ^1^H NMR (CDCl3-d1, 500 MHz): δ/ppm = 8.61 (s, 1H), 7.41 (d, J = 12.5 Hz, 1H), 4.71–4.69 (m, 1H), 4.49 (d, J = 6.5 Hz, 1H), 4.41 (t, J = 6.5 Hz, 2H), 4.33 (d, J = 6.5 Hz, 1H), 4.26 (t, J = 6.0 Hz, 2H), 3.27–3.21 (m, 4H), 3.17 (s, 3H), 3.07 (t, J = 6.0 Hz, 2H), 3.00 (t, J = 6.0 Hz, 2H), 2.46–2.44 (m, 6H), 2.23 (s, 3H), 1.41 (d, J = 6.5 Hz, 3H).

#### 3.2.3. Synthesis of Chitosan-SS-Levofloxacin

LF-SS-COOH (120 mg, 0.2 mmol), EDC (58 mg, 0.3 mmol) and NHS (35 mg, 0.3 mmol) were dissolved in 10 mL of anhydrous DMSO and stirred for 12 h. Chitosan (CS, 100 mg) was dissolved in 30 mL deionized water and stirred in room temperature. In the above reaction solution, the solution was added dropwise, and the mixture was stirred overnight. The resultant solution was dialyzed (MWCO 3.5 kDa) against aqueous NaCl solution (0.1 M) for 48 h and then against deionized water for another 48 h, followed by lyophilization (Yang et al., 2018). The synthesis of CS-SS-LF conjugate was depicted in Figure 10.

Fourier transform infrared spectroscopy (FTIR) and ^1^H NMR (D_2_O/DMSO-d_6_) spectrometry were used to determine the chemical structure of CS-SS-LF.

A UV–vis measurement was used to determine the LF content of micelles. The following formula was used to calculate the LF content:

LF content (wt. %) = (weight of LF/weight of CS-SS-LF) × 100.

### 3.3. Preparation and characterization of Chitosan-SS-Levofloxacin micelles

A total of 10 mg CS-SS-LF conjugation was dispersed in 10 mL DMSO. Stirring the solution for 5 h and dialyzing it against deionized water (MWCO 3.5 kDa) followed. After that, micelles solution was filtered through a microporous membrane with a pore size of 0.45 μM.

A Dynamic Light Scattering (DLS) method (Malvern Zatasizer NANOZS90, Malvern, United Kingdom) was used to determine the average particle size and zeta potential of CS-SS-LF micelles. The morphology of the CS-SS-LF micelles was studied using a field emission scanning electron microscope 163 with an accelerating voltage of 10 kV by Hitachi.

### 3.4. Physical stability of Chitosan-SS-Levofloxacin micelles

DLS was used to measure the diameter and PDI changes of CS-SS-LF micelles after incubation in PBS (pH 7.4) for 6 days.

### 3.5. Reduction-triggered release of levofloxacin *in vitro*

Assaying drug release from LF micelles (CS-SS-LF) in the presence or absence of a 10 mM Na_2_S was conducted in a dialysis tube (MWCO 3.5 kDa) under 100 rpm shaking at 37°C in PBS (10 mM, pH 7.4) containing 10% FBS. The release of drugs was measured using 1.0 mL of CS-SS-LF micelles (1 mg/mL) dispersion dialysis against 30 mL of a corresponding medium. For every desired time interval, 2.0 mL of the release medium was removed and replaced with equal quantities of fresh medium. UV–vis absorption spectrum was used to determine the drug concentration. The data are presented as mean ± SD (*n* = 3).

### 3.6. Antibacterial activity

To investigate the antibacterial activity of this micelles *in vitro*, a two-fold dilution method was employed to determine the minimum inhibitory concentration (MIC) of CS-SS-LF micelles against *Salmonella*, *P*. *aeruginosa*, and *S*. *aureus*. To obtain working cultures, 50 μL of TSB medium was added in a 96-well plate, then 50 μL of bacterial suspensions (*Salmonella*, *P*. *aeruginosa*, and *S*. *aureus*, respectively) was inoculated in each well to adjust the final cell density to approximately 1.0 × 10^5^ CFU/mL. The bacteriostatic agents (CS-SS-LF micelles and LF) was diluted successively and then added to the prepared bacterial suspensions. The concentration of bacteriostatic agent corresponding to wells without bacterial precipitation was the MIC.

Similarly, with the addition of different concentrations of CS-S-S-LF micelles, the bacteria samples (0.4 OD_600_, 0.1 mL) were well mixed with 3.9 mL tryptone soya broth (TSB). At intervals, the OD_600_ was monitored after shaking the mixtures at 37°C.

### 3.7. Antibiofilm activity

For the formation of biofilms, 100 μL of bacterial TSB solutions (~10^8^ CFU/mL) were cultivated in 96-well plates for 24 h at 37°C. After removing non-adhered bacteria, the plate was washed with PBS three times. Afterward, 100 μL TSB containing CS-SS-LF micelles, LF and CS were incubated at 37°C with the existing biofilm. The biofilms were incubated with TSB only as blank control. Each treatment was divided into six parallel wells. As previously reported (Zhang et al., 2013), biofilm mass (Crystal violet staining assay) and viable cells (MTT assay) were evaluated.

As previously described (Mu et al., 2016), *Salmonella* biofilms were grown on glass coverslips placed at the bottom of 12-well plates. The coverslips were washed, and the residual biofilms treated as before.

In order to prepare the SEM sample, *Salmonella* strains were pre-incubated with TSB, CS, LF and CS-SS-LF micelles, followed by removal of the medium, followed by fixing with 4% glutaraldehyde in PBS for 2 h. After that, 40, 50, 70, 90, and 100% ethanol was used to dehydrate the samples. Supercritical CO_2_ drying was then used to dry the bacterial biofilms. SEM analysis was performed on the dried samples after they were plated with platinum.

### 3.8. Cytotoxic activity of Chitosan-SS-Levofloxacin micelles

MTT assays were used to assess the cytotoxicity of CS-SS-LF micelles. RPMI 1640 medium containing 10% FBS was used to culture RAW 264.7 and buffalo rat liver 3A (BRL-3A) cells (6 × 10^3^ cells/well), which were incubated at 37°C with 5% CO_2_ for 24 h. Following that, CS-SS-LF micelles were added to the cells for 24 h in different concentrations. Afterwards, cells were further incubated for 4 h with 20 μL MTT solution. Blue violet crystal formazan was dissolved in 100 μL DMSO. The absorbance was measured at 570 nm using a microplate reader with PBS as a blank control.

The hemolysis assay was performed with CS-SS-LF micelles to check its compatibility with blood. Briefly, red blood cells (RBC) solution (2% w/w) of 0.1 mL was added to 1.9 ml of the solution of CS-SS-LF micelles (250 μg/ml). Deionized water was used as positive controls (PC), while PBS was used as negative controls (NC). After 3 h incubation at 37°C, the samples were centrifuged at 6500 rpm for 10 min, and then 200 μL supernatant was removed carefully and the optical density was measured at 545 nm by a microplate reader. The hemolytic ratio (%) was calculated based on the formula:


$$Hemolysis\;ratio\left(\%\right)=\left(OD_{sample}-OD_{NC}\right)/\left(OD_{PC}-OD_{NC}\right)\times 100$$

### 3.9. *In vivo* activity evaluation

The experiment was conducted using female Kunming mice (6 weeks old). *Salmonella* was injected intraperitoneally into mice at a dose of about 1 × 10^6^ CFU per animal (Zhi et al., 2016). Three times a day for 3 days, mice received subcutaneous injections of CS-SS-LF micelles, LF, CS, or PBS (10 mg/kg of CS-SS-LF micelles, n = 5). An aseptic procedure was used to remove the liver, spleen, and kidneys from the mice, which were homogenized with sterile saline solution in a final volume of 1 mL. By plating serial dilutions of the cultures onto TSB agar, CFUs were counted.

### 3.10. Statistical analysis

Plots and statistical analyses were carried out using the software GraphPad Prism 7.01. Experimental data were expressed as mean ± standard deviation (SD).

## 4. Conclusion

In this study, a H_2_S-responsive amphiphilic chitosan and levofloxacin conjugate (CS-SS-LF), which can self-assemble to micelles was successfully developed. The desirable H_2_S-sensitivity of CS-SS-LF micelles were verified *via in vitro* drug release study in the presence of Na_2_S. Furthermore, the CS-SS-LF micelles showed stronger capacity of biofilm eradication and decreased organ bacterial counts in mice. As an novel antibiotic delivery vehicles, CS-SS-LF micelles are a viable approach for targeting bacteria and releasing antibiotics upon exposure to the infection microenvironment, thus triggering the release of antibiotics. Accordingly, our presented H_2_S-responsive strategy not only provides a robust way to solve the contradiction of simultaneous transport and premature catabolism of the traditional drug system but also offers an opportunity for utilizing infection microenvironment as a trigger to develop on-demand antibacterial agents.

## Data availability statement

The original contributions presented in the study are included in the article/Supplementary material, further inquiries can be directed to the corresponding authors.

## Ethics statement

This study was performed with the approval of the Experimental Animal Manage Committee (EAMC) of Weifang Medical University. Animals were treated as the guidelines of EAMC.

## Author contributions

CL and YX conceived the experiments and wrote and revised the manuscript. CL, WL, XQ, SL, CN, and JG carried out the experiments and performed data analysis. All authors contributed to the article and approved the submitted version.

## Funding

This research was funded by the Natural Science Foundation of Shandong Province (Grant No. ZR2021QC178).

## Conflict of interest

The authors declare that the research was conducted in the absence of any commercial or financial relationships that could be construed as a potential conflict of interest.

## Publisher’s note

All claims expressed in this article are solely those of the authors and do not necessarily represent those of their affiliated organizations, or those of the publisher, the editors and the reviewers. Any product that may be evaluated in this article, or claim that may be made by its manufacturer, is not guaranteed or endorsed by the publisher.

## References

[ref1] Alvarez-Lorenzo C. Garcia-Gonzalez C. A. Bucio E. Concheiro A. (2016). Stimuli-responsive polymers for antimicrobial therapy: drug targeting, contact-killing surfaces and competitive release. Expert Opin. Drug Deliv. 13, 1109–1119. doi: 10.1080/17425247.2016.1178719, PMID: 27074830

[ref2] Barton B. C. Jones T. F. Vugia D. J. Long C. Marcus R. Smith K. . (2011). Deaths associated with bacterial pathogens transmitted commonly through food: foodborne diseases active surveillance network (FoodNet), 1996-2005. J. Infect. Dis. 204, 263–267. doi: 10.1093/infdis/jir263 21673037

[ref3] Chambers H. F. Deleo F. R. (2009). Waves of resistance: *Staphylococcus aureus* in the antibiotic era. Nat. Rev. Microbiol. 7, 629–641. doi: 10.1038/nrmicro2200, PMID: 19680247 PMC2871281

[ref4] Chen M. Xie S. Wei J. Song X. Ding Z. Li X. (2018). Antibacterial micelles with vancomycin-mediated targeting and pH/lipase-triggered release of antibiotics. ACS Appl. Mater. Interfaces 10, 36814–36823. doi: 10.1021/acsami.8b16092, PMID: 30298721

[ref5] Christopher C. M. Patrick B. David A. P. Samuel I. M. (2001). Chapter 17–nontyphoidal salmonellosis. Clin. Infect. Dis. 32, 263–269. doi: 10.1086/318457 11170916

[ref6] Dissaya P. Li Z. Sage O. Santosh A. Marygorret O. Kenneth V. (2011). Bacterial toxin-triggered drug release from gold nanoparticle-stabilized liposomes for the treatment of bacterial infection. J. Am. Chem. Soc. 133, 4132-4139. doi: 10.1021/ja111110e 21344925 PMC3062754

[ref7] El-Refaie K. Worley S. D. Roy B. (2007). The chemistry and applications of antimicrobial polymers: a state-of-the-art review. Biomacromolecules 8, 1359–1384. doi: 10.1021/bm061150q 17425365

[ref8] Francolini I. Norris P. Piozzi A. Donelli G. Stoodley P. (2004). Usnic acid, a natural antimicrobial agent able to inhibit bacterial biofilm formation on polymer surfaces. Antimicrobial Agents Chemother. 48, 4360–4365. doi: 10.1128/AAC.48.11.4360-4365.2004, PMID: 15504865 PMC525405

[ref9] Gao W. Chen Y. Zhang Y. Zhang Q. Zhang L. (2018). Nanoparticle-based local antimicrobial drug delivery. Adv. Drug Deliv. Rev. 127, 46–57. doi: 10.1016/j.addr.2017.09.015, PMID: 28939377 PMC5860926

[ref10] Ghosh S. Wu V. Pernal S. Uskoković V. (2016). Self-setting calcium phosphate cements with tunable antibiotic release rates for advanced antimicrobial applications. ACS Appl. Mater. Interfaces 8, 7691–7708. doi: 10.1021/acsami.6b01160, PMID: 26958867 PMC5002010

[ref11] Hajji S. Younes I. Rinaudo M. Jellouli K. Nasri M. (2015). Characterization and in vitro evaluation of cytotoxicity, antimicrobial and antioxidant activities of Chitosans extracted from three different marine sources. Appl. Biochem. Biotechnol. 177, 18–35. doi: 10.1007/s12010-015-1724-x, PMID: 26150381

[ref12] Hudson S. M. (2003). Review of chitosan and its derivatives as antimicrobial agents and their uses as textile chemicals. J. Macromolecul. Sci. 43, 223–269. doi: 10.1081/MC-120020161

[ref13] Jalvandi J. White M. Gao Y. Truong Y. B. Padhye R. Kyratzis I. L. (2017). Polyvinyl alcohol composite nanofibres containing conjugated levofloxacin-chitosan for controlled drug release. Mater. Sci. Eng. C Mater. Biol. Appl. 73, 440–446. doi: 10.1016/j.msec.2016.12.112, PMID: 28183630

[ref14] Lin D. Yan M. Lin S. Chen S. (2014). Increasing prevalence of hydrogen sulfide negative salmonella in retail meats. Food Microbiol. 43, 1–4. doi: 10.1016/j.fm.2014.04.010, PMID: 24929875

[ref15] Meng-Hua X. Ya-Juan L. Yan B. Xian-Zhu Y. Bing H. Jun W. (2012a). Bacteria-responsive multifunctional nanogel for targeted antibiotic delivery. Adv. Mater. 24, 6175–6180. doi: 10.1002/adma.201202847 22961974

[ref16] Meng-Hua X. Yan B. Xian-Zhu Y. Yu-Cai W. Baolin S. Jun W. (2012b). Lipase-sensitive polymeric triple-layered nanogel for "on-demand" drug delivery. J. Am. Chem. Soc. 134, 4355–4362. doi: 10.1021/ja211279u 22304702

[ref17] Mu H. Tang J. Liu Q. Sun C. Wang T. Duan J. (2016). Potent antibacterial nanoparticles against biofilm and intracellular bacteria. Sci. Rep. 6:18877. doi: 10.1038/srep18877, PMID: 26728712 PMC4700437

[ref18] Nichterlein T. Bornitz F. Kretschmar M. Hof H. (1998). Successful treatment of murine listeriosis and salmonellosis with levofloxacin. J. Chemother. 10, 313–319. doi: 10.1179/joc.1998.10.4.313, PMID: 9720471

[ref19] Ning L. G. Kang E. T. Wang Y. B. Hu X. F. Xu L. Q. (2018). Recent developments in controlled release of antibiotics. Curr. Pharm. Des. 24, 911–925. doi: 10.2174/1381612824666180315094947 29542409

[ref20] Pal S. Ramu V. Taye N. Mogare D. G. Yeware A. M. Sarkar D. . (2016). GSH induced controlled release of levofloxacin from a purpose-built prodrug: luminescence response for probing the drug release in Escherichia coli and *Staphylococcus aureus*. Bioconjug. Chem. 27, 2062–2070. doi: 10.1021/acs.bioconjchem.6b00324, PMID: 27506475

[ref21] Prost E. Riemann H. (1967). Food-borne salmonellosis. Annu. Rev. Microbiol. 21, 495–528. doi: 10.1146/annurev.mi.21.100167.002431 4860266

[ref22] Silva L. N. Zimmer K. R. Macedo A. J. Trentin D. S. (2016). Plant natural products targeting bacterial virulence factors. Chem. Rev. 116, 9162–9236. doi: 10.1021/acs.chemrev.6b00184 27437994

[ref23] Stryjewski M. E. Corey G. R. (2014). Methicillin-resistant *Staphylococcus aureus*: an evolving pathogen. Clin. Infect. Dis. 58:S10. doi: 10.1093/cid/cit613 24343827

[ref24] Wang Z. Bai H. Lu C. Hou C. Qiu Y. Zhang P. . (2019). Light controllable chitosan micelles with ROS generation and essential oil release for the treatment of bacterial biofilm. Carbohydr. Polym. 205, 533–539. doi: 10.1016/j.carbpol.2018.10.095, PMID: 30446137

[ref25] Yang Y. Zhao Y. Lan J. Kang Y. Zhang T. Ding Y. . (2018). Reduction-sensitive CD44 receptor-targeted hyaluronic acid derivative micelles for doxorubicin delivery. Int. J. Nanomedicine Volume 13, 4361–4378. doi: 10.2147/IJN.S165359, PMID: 30100720 PMC6065576

[ref26] Yannan Z. Trewyn B. G. Slowing I. I. Victor S.-Y. L. (2009). Mesoporous silica nanoparticle-based double drug delivery system for glucose-responsive controlled release of insulin and cyclic AMP. J. Am. Chem. Soc. 131:8398. doi: 10.1021/ja901831u 19476380

[ref27] Younes I. Hajji S. Frachet V. Rinaudo M. Jellouli K. Nasri M. (2014). Chitin extraction from shrimp shell using enzymatic treatment. Antitumor, antioxidant and antimicrobial activities of chitosan. Int. J. Biol. Macromol. 69, 489–498. doi: 10.1016/j.ijbiomac.2014.06.013, PMID: 24950313

[ref28] Zaidi S. Misba L. Khan A. U. (2017). Nano-therapeutics: a revolution in infection control in post antibiotic era. Nanomedicine 13, 2281–2301. doi: 10.1016/j.nano.2017.06.015 28673854

[ref29] Zhang A. Mu H. Zhang W. Cui G. Zhu J. Duan J. (2013). Chitosan coupling makes microbial biofilms susceptible to antibiotics. Sci. Rep. 3:3364. doi: 10.1038/srep03364, PMID: 24284335 PMC3842539

[ref30] Zhi L. Qing Z. Xiaoyan X. Xin S. Ying Z. Fei D. . (2016). Pyroptosis of salmonella typhimurium-infected macrophages was suppressed and elimination of intracellular bacteria from macrophages was promoted by blocking QseC. Sci. Rep. 6:37447. doi: 10.1038/srep37447 27853287 PMC5112599

