# Supplementary material for: Hydrogen sulfide-sensitive Chitosan-SS-Levofloxacin micelles with a high drug content: Facile synthesis and targeted Salmonella infection therapy
Source: Front Microbiol. 2022 Dec 22;13:1088153. doi: 10.3389/fmicb.2022.1088153 (PMC9813597; doi:10.3389/fmicb.2022.1088153)
Supplement: Supplementary file 1 [file Data_Sheet_1.docx]

**Supplementary Information for:**

**Hydrogen sulfide-Sensitive Chitosan-SS-Levofloxacin Micelles with a High Drug Content: Facile Synthesis and Targeted *Salmonella* Infection Therapy**

**Chunbo Lu^a，b^*, Wenhui Lu^a^, Xiantao Qin^a^, Shuyi Liang^a^, Congmin Niu^a^, Jiayi Guo^a^, Yujie Xu^b,*^**

^a^ Weifang Med Univ, Sch Biosci & Technol, Key Lab Biol Med Univ Shandong Prov, Baotong Rd, Weifang 261053, Shangdong, Peoples R China;

^b^ College of Chemistry & Pharmacy, Shaanxi Key Laboratory of Natural Products & Chemical Biology, Northwest A&F University, Yangling 712100, Shaanxi, China;

*** Correspondence:** Chunbo Lu [Lucb163@163.com](mailto:Lucb163@163.com);

Yujie Xu 286265978@qq.com.

Table 1. MIC of CS-SS-LF micelles and free LF against different pathogenic bacteria.


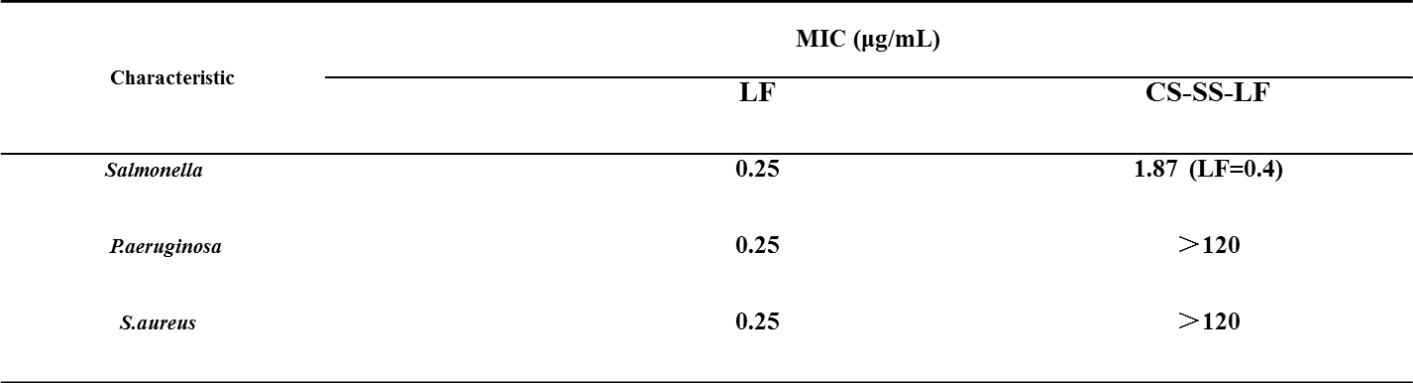


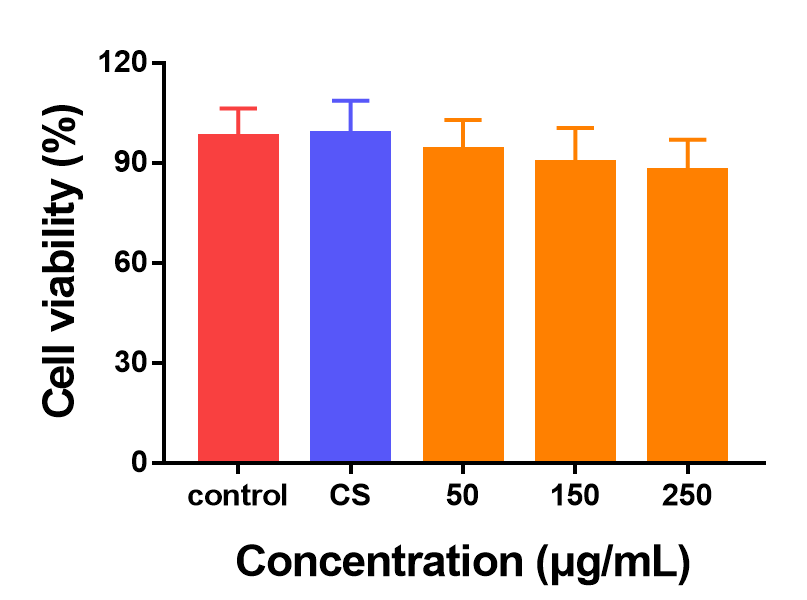


Figure S1. Relative viabilities of BRL-3A cells after being exposed to CS-SS-LF micelles with different concentrations for 24 h.
